# Distinct Adverse Reactions to mRNA, Inactivated Virus, and Adenovirus Vector COVID-19 Vaccines: Insights from a Cohort Study on Atopic and Non-Atopic Subjects in Brazil

**DOI:** 10.3390/vaccines12040408

**Published:** 2024-04-12

**Authors:** Laura Alves Ribeiro Oliveira, Alessandro Sousa Correa, Thiago Alves de Jesus, Miguel Junior Sordi Bortolini, Ernesto Akio Taketomi, Rafael de Oliveira Resende

**Affiliations:** 1 Laboratory of Allergy and Clinical Immunology, Institute of Biomedical Sciences, Federal University of Uberlândia, Uberlândia 38405-317, Brazil; laura.alves@ufu.br (L.A.R.O.); alessandro.correa@ufu.br (A.S.C.); thiago498.tadj@ufu.br (T.A.d.J.); taketomi@ufu.br (E.A.T.); 2 Laboratory of Translational Immunology, Health and Sports Sciences Center, Federal University of Acre, Rio Branco 69920-900, Brazil; miguel.bortolini@ufac.br; 3 Laboratory on Thymus Research, Oswaldo Cruz Institute, Fiocruz, Rio de Janeiro 21040-900, Brazil

**Keywords:** adverse effects, atopic, vaccine, allergy, COVID-19

## Abstract

The emergence of COVID-19 caused by SARS-CoV-2 prompted an unprecedented global response to develop vaccines at an accelerated pace. Messenger RNA (mRNA) and adenovirus vector vaccines emerged as the frontrunners in global immunization efforts, significantly reducing hospitalization, severity, and mortality, supplemented by inactivated virus-based vaccines in developing countries. However, concerns regarding adverse effects, including allergic reactions, have been raised. This study aimed to investigate the adverse effects following COVID-19 vaccination, particularly in atopic and non-atopic individuals. A cohort of 305 volunteers receiving BNT162, ChAdOx1, or CoronaVac vaccines were assessed based on a Skin Prick Test (SPT), specific IgE levels, and clinical history of asthma and rhinitis. Adverse effects were self-reported and scored across the different vaccination shots. The results indicated a notable presence of mild adverse effects following the first and third doses, regardless of vaccine type. ChAdOx1 recipients experienced more adverse effects compared to those receiving BNT162 and CoronaVac, including headaches, muscle pain, fever, chills, nausea, and flu-like symptoms. Atopic individuals receiving ChAdOx1 reported more adverse effects, such as muscle pain, fever, and chills, compared to non-atopic individuals. Conversely, headaches were more frequently reported in non-atopic individuals receiving BNT162 compared to atopic individuals. No anaphylaxis or allergic reactions were reported, indicating valuable evidence supporting the safety of COVID-19 vaccination in individuals with respiratory allergies. This study highlights the importance of understanding vaccine-related adverse effects, particularly in vulnerable populations, to inform vaccination strategies and address safety concerns in global immunization campaigns.

## 1. Introduction

Cases of COVID-19, caused by the SARS-CoV-2 virus, were initially reported in December 2019 in Wuhan, China, and subsequently spread globally [1]. Given the swift increase in infection cases, the World Health Organization (WHO) declared it a Public Health Emergency of International Concern (PHEIC) in January 2020 [2], and this status remained until May 2023. During this stage, several vaccine candidates started to be developed and tested at a never-before-seen scale to mitigate severe cases [3]. Also, considering the escalating global SARS-CoV-2 infection rates, international alliances were formed to promptly organize resources in order to accelerate vaccine production [4,5].

In addition to conventional technologies, the COVID-19 pandemic catalyzed advancements in novel vaccine platforms [6]. The BNT162 mRNA vaccine, developed by Pfizer–BioNTech, and the ChAdOx1 adenoviral vector vaccine, developed by the University of Oxford–AstraZeneca, were the initial vaccines endorsed by the World Health Organization (WHO) for emergency deployment [7]. They have predominantly been adopted worldwide, complemented by the administration of other vaccines, such as the inactivated virus vaccine CoronaVac (Sinovac), which has primarily been distributed in low- and middle-income nations [8,9]; together, they markedly reduced the infection rates, severity, and mortality, preventing millions of deaths [10].

The activation of the immune system provided by T regulatory cells and balance between Type 1 and Type 2 immune responses is crucial for individual protection induced by both natural infections and vaccination. Clinical trials and life-real studies demonstrated that the antibody response induced by the COVID-19 vaccines involves the production of IgG, particularly IgG1 and IgG3, and Type 1 cytokines, such as IFN-γ and TNF-α, which is mediated by CD4^+^ and CD8^+^ T cells [11,12,13].

A wide spectrum of adverse effects has been documented across all the COVID-19 vaccine formulations, including vaccine-related symptoms or allergic reactions. These effects span from common mild symptoms including local pain, fever, and headaches to anaphylaxis, which represents the most severe form of reaction [14]. Although rare, cases of allergic reactions to vaccines have been the focus of many studies [15]. A hypothesis that individuals with pre-existing respiratory allergy might have a higher likelihood of developing anaphylactic or non-anaphylactic responses post-COVID-19 vaccination has been considered [16,17,18]. A tolerance study with the BNT162 mRNA vaccine conducted by Nittner-Marszalska et al. in 2021 [19] reported a higher duration of systemic adverse effects in allergic patients in addition to a higher frequency of local reactions, headaches, and nausea, as was described elsewhere [20]. An exacerbation of the clinical picture of asthma has been reported after vaccination with an inactivated virus-based COVID-19 vaccine [21], but there are limited studies with a large number of patients focused on respiratory allergies as a side effect. Although some of these observational studies have suggested an association between a previous history of allergy and adverse effects related to COVID-19 vaccination, the mechanism underlying these reactions is still not very clear and it should be explored.

The term atopy is employed to denote the genetic tendency to produce IgE against a small quantity of allergens, typically harmless environmental proteins, followed by a Type 2 immune response [22]. This condition may predispose individuals to the development of allergic diseases, including gastrointestinal disorders, atopic dermatitis, asthma, and rhinitis, among others. A diagnosis of atopy relies on an assessment of specific IgE and/or positivity to a Skin Prick Test (SPT). However, the presence of IgE itself does not indicate an allergy, as allergic responses may also be mediated by other immune components, such as specific IgG, innate immune cells, C3a/C5a complement anaphylatoxins, and cytokines. Consequently, in clinical practice, individuals with an allergy may be categorized as non-atopic if conventional diagnostic tests for IgE yield negative outcomes [23].

Asthma and rhinitis symptoms may be triggered by inhalant allergens from pollen and non-seasonal sources, such as house dust mites, mold, and pets, which elicit both local and systemic immune-mediated inflammation [24]. The most recent classifications for asthma and rhinitis endotypes include upstream and downstream biomarkers, based on omics signatures for IgE, cytokine profiles, neutrophil/basophil populations, and other inflammatory factors [24,25,26]. However, although this approach is reliable for endotype and phenotype differentiation, the specific IgE assessment still represents the state of the art for atopy diagnosis.

In atopic patients with respiratory allergy, it has been demonstrated that specific IgE can downregulate Type 1 immune response to pathogens. A lack of IgG against infectious agents can be substantially attributed to IgE-mediated immune responses in atopic subjects with higher levels of IgE against *Dermatophagoides farinae* and *D. pteronyssinus*, two of the main house dust mite species that cause respiratory allergy [27]. In the context of COVID-19, an observational study has elucidated that atopic subjects with specific IgE reactivity to house dust mite antigens exhibit a more favorable disease prognosis. This was coupled with the observation of an immune response predominantly orchestrated by interleukin-13 (IL-13), a potent Type 2 cytokine, suggesting a protective immunological mechanism within this population. Notably, there was no evidence of cross-reactivity between these allergens and SARS-CoV-2 spike protein. Furthermore, increased expression of IL-13 was discerned in these patients after mRNA vaccination, while the levels of specific IgE remained elevated compared to non-atopic individuals [28]. To date, investigations into the potential impact of house dust mite-specific IgE on the occurrence of adverse effects following COVID-19 vaccination have yet to be undertaken.

In this study, we aimed to investigate the safety profile of mRNA, inactivated virus, and adenovirus vector-based COVID-19 vaccines in atopic and non-atopic individuals. The rationale for this research stems from the growing concern over potential adverse effects of COVID-19 vaccines, particularly in populations with underlying allergy conditions. By comparing the incidence and severity of adverse events between subjects with or without atopy, we sought to elucidate any differences in vaccine safety profiles based on allergic status. Understanding these differences is crucial for informing vaccination strategies and mitigating risks in vulnerable populations.

## 2. Materials and Methods

### 2.1. Study Design

To investigate if individuals with atopy and a clinical history of respiratory allergy exhibit distinct adverse reactions following COVID-19 vaccination compared to non-atopic individuals, we used a cohort of regular patients, alumni, and hospital staff, who were recruited by an allergy service in Brazil. For atopic/non-atopic classification, they were evaluated for immediate hypersensitivity and specific IgE levels.

The study was based on the Brazilian Vaccination Program for COVID-19 in adults, started in January 2021, with a vaccine schedule consisting of two doses (shot 1 and shot 2, homologous) administered at 8-week intervals for monovalent BNT162 and ChAdOx1 and 28 days for CoronaVac. A heterologous booster dose (shot 3) was included 16 weeks after shot 2. The research was approved by the local ethics committee (Protocol CAAE-50001421.7.0000.5152 CEP/UFU) and informed consent was obtained from all participants prior to their inclusion in the study.

The study was conducted at the Clinics Hospital of the Federal University of Uberlândia, southeastern region of Brazil, with 305 volunteers (18–50 years old) who received BNT162, ChAdOx1, or CoronaVac vaccines between July 2022 and September 2023. Demographic data, including age and sex, along with vaccine-related information, such as the date of the shots, adverse effects, allergic reactions, and type of vaccine administered, were collected as part of the study protocol. The participants were free to report any adverse effects they experienced without limitations or pre-defined options and the answers were categorized. An adverse effect score was determined as the cumulative number of symptoms reported.

Atopic individuals were diagnosed by a positive SPT and/or the presence of house dust mite-specific IgE and those with negative outcomes for both tests were considered non-atopic. The prevalence of rhinitis and asthma was determined by RCAT (Rhinitis Control Assessment Test) and ACT (Asthma Control Test) clinical questionnaires, although these features were not used to determine atopy status. Individuals who were being treated with corticosteroid, antihistamine, or immunosuppressive medication were excluded from the study.

### 2.2. SPT with Airborne Allergens and D. farinae-Specific IgE ELISA

SPT was conducted using house dust mite (*Dermatophagoides farinae*, *D. pteronyssinus*, and *Blomia tropicalis*), dog (*Canis familiaris*), and cat dander (*Felis catus*) allergen extracts (FDA Allergenic, Rio de Janeiro, Brazil) [29]. A wheal diameter ≥ 3 mm after 15 min was considered positive. Serum samples were used to detect *D. farinae*-specific IgE by an indirect ELISA [30]. Briefly, high-affinity microplates were coated with 40 μg/mL *D. farinae* extract for 18 h at 4 °C and blocked with phosphate-buffered saline/0.05% Tween (PBS-T) with 1% Bovine Serum Albumin (BSA). Serum samples (1:2) were diluted in PBS-T-BSA and incubated at 37 °C for 2 h, followed by incubation with biotinylated anti-human IgE and streptavidin–peroxidase conjugate (1:1000) at room temperature. The reaction was developed by adding 0.01 M 2,2′-azino-bis-3-ethylbenzothiazoline-6-sulfonic acid (ABTS) and the absorbance values were determined at 405 nm. The results were expressed as an ELISA index (EI) score, as described elsewhere [31]. EI ≥ 1.2 was considered positive.

### 2.3. Statistical Analysis

The statistical significance of differences based on sex, age, and presence of rhinitis and/or asthma were assessed using Student’s *t*-tests, Chi-square tests, or Fisher tests, when applicable. Differenced among the vaccine shots were compared using Kruskal–Wallis tests followed by the Dunn’s multiple comparison test, with *p* < 0.05 considered to be statistically significant. All statistical analyses were conducted using Prism 9.4 (GraphPad, Boston, MA, USA).

## 3. Results

### 3.1. Atopic and Non-Atopic Groups

The subjects were divided in atopic and non-atopic groups, based on SPT and/or specific IgE outcomes. From the 305 people who joined the study, a slightly higher percentage (54.4%) were atopic (Table 1). Although all the volunteers were 18 to 50 years old, the mean age was 30.7 years in the atopic group and 35.0 years in the non-atopic group. In both the atopic and non-atopic groups, female participants predominated at 62.1% and 73.3%, respectively. Although allergic symptoms were not considered for atopy definition, the vast majority of the atopic group (69.8%) reported rhinitis symptoms and even among the non-atopic participants, we found that 38.2% of the patients had rhinitis symptoms. In addition, in the atopic group, only 0.6% reported asthma whereas 9.6% had respiratory allergies. In the non-atopic group, the number of asthmatics was slightly higher (0.7%), but not when associated with rhinitis (2.8%).

In the SPT, the mean wheal size in the atopic group was 5.5 mm for *D. farinae*, 4.8 mm for *D. pteronyssinus*, and 3.6 mm for *B. tropicalis*, followed by 3.9 mm for cat dander and 3.7 mm for dog extracts. All participants in the non-atopic group had negative results in this test.

Regarding *D. farinae*-specific IgE levels, as expected, the atopic subjects reached higher EI values compared to the non-atopic group (2.4 vs. 0.7, *p* < 0.0001). The positivity threshold was set to 1.2 to avoid biased results.

### 3.2. Adverse Effects of the COVID-19 Vaccines

**Figure 1:**
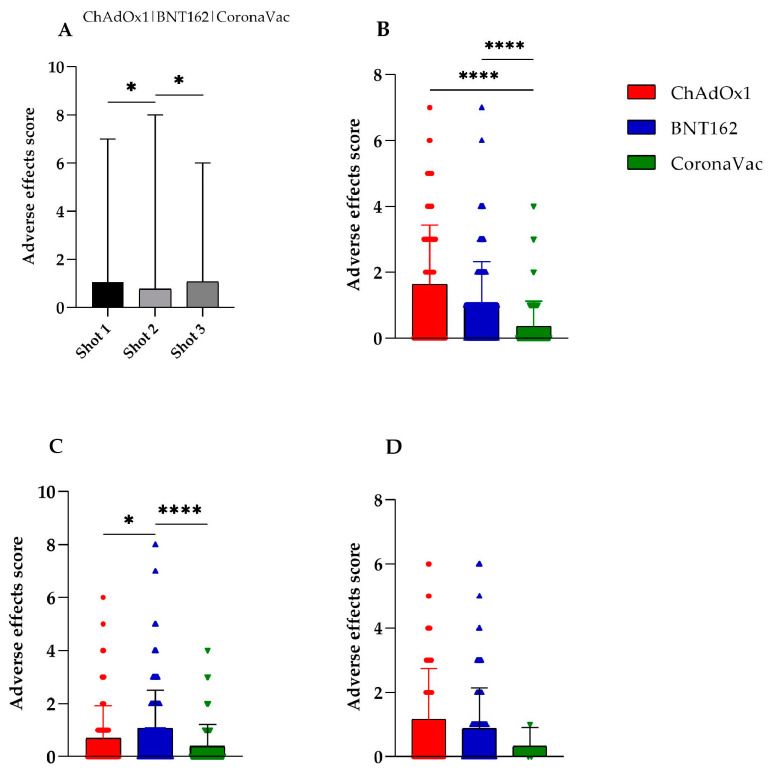
Comparison of the adverse effect scores of the BNT162, ChAdOx1, and CoronaVac vaccines for COVID-19 among (**A**) shots 1, 2, and 3; (**B**) shot 1 only; (**C**) shot 2 only; (**D**) shot 3 only. * *p* < 0.05; **** *p* < 0.0001.

All individuals reported at least one adverse effect following COVID-19 vaccination, although they were predominantly mild. No allergic, anaphylactic, or other severe reactions were reported. All adverse effects were compared over the three shots. The first and third shots had higher adverse effect mean scores (1.04 for shot 1 and 1.06 for shot 3) compared to the second shot (0.76, *p* < 0.05) when all vaccines were analyzed (Figure 1A).

We decided to compare the different vaccines independently for each shot to determine if any vaccine had a greater impact on the overall adverse effects data. In the first shot, both the BNT162 and ChAdOx1 vaccines showed no differences between the mean scores (1.6 vs. 1.05, respectively; *p* = 0.3851). Despite this, both had higher mean adverse effects scores compared to CoronaVac (0.36, *p* < 0.0001) (Figure 1B).

In the second shot, BNT162 elicited a higher mean score (1.07) compared to ChAdOx1 (0.69, *p* < 0.05) and CoronaVac (0.39, *p* < 0.0001). However, there was no difference between the mean adverse effect score of ChAdOx1 and CoronaVac (*p* = 0.22) (Figure 1C). Surprisingly, no statistical difference was found between the mean adverse effect scores for the third shot for all vaccines. Nevertheless, it is noteworthy that there was a subtle decrease in subjects who received CoronaVac (Figure 1D).

**Figure 2:**
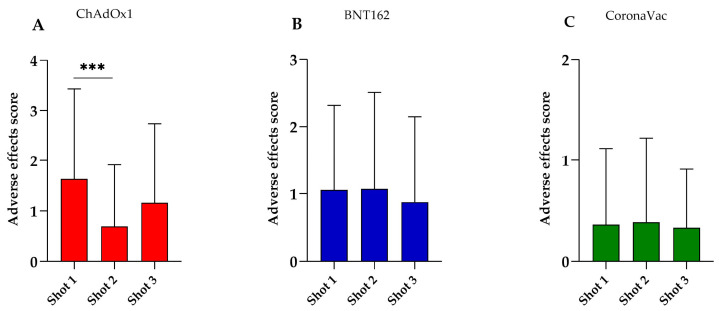
Adverse effect score distribution of (**A**) ChAdOx1, (**B**) BNT162, and (**C**) CoronaVac vaccines for COVID-19. The score was calculated as the cumulative number of adverse effects, ranging from 1 to 4. *** *p* < 0.001.

Therefore, we aimed to investigate if a specific shot of each of these vaccines had a greater impact on the mean values of adverse effects. Figure 2 illustrates the adverse effects after the administration of each vaccine. Notably, only the ChAdOx1 vaccine induced a higher incidence of adverse effects in the first shot (1.63) compared to the second shot (0.69, *p* < 0.001). In contrast, there was no significant difference when compared to the third shot (1.16, *p* = 0.1655), and the same was observed between the second and third shots (*p* = 0.1575) (Figure 2A). On the other hand, the BNT162 and CoronaVac vaccines did not show significant differences in the average symptom score across the vaccination shots (Figure 2B and Figure 2C, respectively).

We also aimed to investigate differences in symptoms associated with the vaccines; then, we focused our analysis on the first shot, which had the largest sample of people available. We assessed the following symptoms: local pain at the injection site, headache, muscle pain, fatigue, fever, chills, enlarged lymph nodes, nausea, and flu-like symptoms (Table 2).

The patients who received the BNT162 vaccine reported higher levels (49.6%) of local pain compared to those who received the ChAdOx1 (31.9%) and CoronaVac (16.5%) vaccines (*p* < 0.05). Similarly, the ChAdOx1 vaccine was associated with a greater number of symptoms compared to CoronaVac (*p* < 0.05) (Figure S1).

Regarding headaches, the ChAdOx1 (22.7%) vaccine reached a higher number of cases than the BNT162 (10.5%) and CoronaVac (3.5%) vaccines (*p* < 0.05).

For muscle pain, a higher prevalence was observed in the subjects vaccinated with ChAdOx1 (30.9%) compared to the groups receiving BNT162 (11.4%) and CoronaVac (5.9%) (*p* < 0.001). However, no significant differences were observed between the latter two groups regarding this symptom (*p* = 0.22) (Figure S1).

Regarding fatigue, differences were only observed between the BNT162 (16.3%) and CoronaVac (5.9%) vaccines (*p* < 0.05), indicating that BNT162 triggered this symptom more frequently. In subjects who reported fever, a higher frequency was observed with the ChAdOx1 (25.8%) vaccine compared to BNT162 (8.1%) and CoronaVac (2.3%) (*p* < 0.001).

A higher prevalence of chills was evidenced in the ChAdOx1 vaccine (12.4%) compared to the BNT162 (3.2%) and CoronaVac vaccines (*p* < 0.01). However, when comparing CoronaVac and BNT162, no significant differences were identified in the averages of subjects with this adverse effect (*p* = 0.09).

In terms of nausea, the subjects who received the ChAdOx1 (11.3%) vaccine exhibited a higher incidence compared to those vaccinated with BNT162 (1.6%) and CoronaVac (*p* < 0.01).

Additionally, a more pronounced prevalence of flu-like adverse effects was noted when comparing the ChAdOx1 (9.3%) and CoronaVac (2.3%) (*p* < 0.05) vaccines. Nevertheless, no significant disparities were evident between BNT162 and ChAdOx1 (*p* = 0.06) or between BNT162 and CoronaVac (*p* = 0.38) (Figure S1). Moreover, there were no detectable differences among subjects who received the three vaccines in terms of lymph node enlargement. Table 2 summarizes the frequency of adverse effects reported for each vaccine.

### 3.3. Adverse Effects of the COVID-19 Vaccines in Atopic and Non-Atopic Subjects

**Figure 3:**
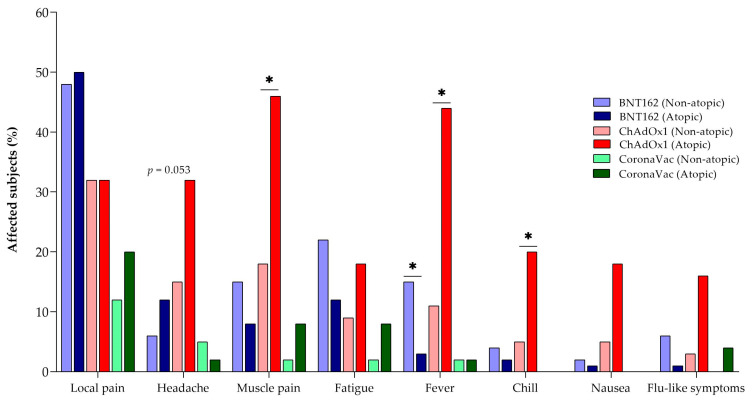
Main adverse events associated with the administration of the first dose of the BNT162, CoronaVac, and ChAdOx1 vaccines in the non-atopic and atopic groups. The colors indicate the type of vaccine, and the intensity of the relative color indicates the non-atopic (lighter) or atopic (darker) groups. * *p* < 0.05.

After analyzing the differences in adverse effects of the different vaccines, our investigation focused on the presence of significant differences between atopic and non-atopic individuals based on the first shot data. When considering all vaccines, we found no significant differences between the atopic and non-atopic groups for any of the adverse effects reported (Table S1). We also conducted an analysis based on the type of vaccine administered and reclassified individuals into atopic and non-atopic groups to assess whether there was a statistically significant difference in the presence of adverse effects for the same vaccine (Figure 3, Tables S2–S4). When analyzing local pain, headache, fatigue, nausea, and flu-like symptoms, no statistical differences were observed between the atopic and non-atopic groups, although there was a tendency (*p* = 0.053) for a greater adverse effect of headaches in atopic individuals immunized with ChAdOx1 when compared to non-atopic individuals (Figure 3, Table S3).

When analyzing muscle pain, fever, and chills as a result of the ChAdOx1 vaccine, these symptoms were more common in atopic individuals than in non-atopic individuals (*p* < 0.05). In contrast, when the BNT162 vaccine was studied, non-atopic individuals were found to have fever more frequently than atopic individuals (*p* < 0.05) (Figure 3, Table S2).

## 4. Discussion

The present study aimed to investigate the adverse effects of three COVID-19 vaccines (ChAdOx1, BNT162, and CoronaVac) in individuals with or without atopy, focused on respiratory allergies (allergic rhinitis and/or asthma). We assessed self-reported participant information to elucidate the common adverse effects and administered questionnaires (RCAT and ACT) to investigate allergic symptoms; in addition, we performed SPT and *D. farinae*-specific IgE quantification, which is one of the most important allergen sources that causes respiratory allergic diseases in tropical and sub-tropical regions. All atopic individuals were positive in the SPT and/or for specific IgE, and non-atopic individuals were negative for both tests. The total IgE level was not assessed in this study because in countries where there are a large number of cases of helminth parasitosis, such as Brazil, total IgE results may not be truly representative due to influence of *Ascaris lumbricoides*, *Strongyloides stercoralis*, and *Toxocara* spp. allergens [32].

The results demonstrated that most subjects, regardless of being atopic, showed no adverse effects after the first and third doses, independent of the vaccine applied. When we evaluated the vaccinations separately, ChAdOx1 and BNT162 revealed a similar number of adverse effects after the first shot compared to CoronaVac. The study did not analyze the data from the third shot because during the collection period, the number of participants complying with the second shot was smaller. Thus, we used the data from the first shot for the examination of the side effects typically reported following immunization. In general, individuals who received a first shot of the ChAdOx1 vaccine exhibited more side effects, such as headache, muscle pain, fever, chills, nausea, and flu-like symptoms, compared to those who received the BNT162 vaccine. Local pain, fever, fatigue, and myalgia, although causing discomfort, are commonly observed following vaccinations and are typically not concerning [33]. A cross-sectional study analyzed the self-reported side effects of the BNT162 vaccine, with similar results; the subjects mostly reported headaches, chills, fatigue, and fever as systemic symptoms [34]. In another cross-sectional study conducted with healthcare professionals, with an average age of 31 years and who were vaccinated with ChAdOx1, a higher frequency of side effects was also observed after the first shot. The predominant symptoms included headache, fatigue, fever, muscle pain, chills, and nausea, along with other symptoms like joint pain, loss of taste, and loss of appetite [35]. In our study, the most commonly reported side effect in both ChAdOx1 and BNT162 vaccine groups was local pain. In addition, the participants did not report any allergic symptoms or anaphylaxis that could be linked to the vaccines. In other studies, including phase II clinical trials, more than 80% of the participants also reported the same effect after vaccination [36,37].

To investigate whether individuals with atopic conditions such as allergic rhinitis and/or asthma experience more prominent side effects than non-atopic individuals after vaccination and given that individuals who received the ChAdOx1 vaccine showed significantly more adverse effects compared to those who received BNT162 and CoronaVac, we stratified the effects by group and vaccine administered. Atopic individuals who received ChAdOx1 reported the most adverse effects such as muscle pain, fever, and chills compared to non-atopic individuals. However, in another study evaluating the BNT162 vaccine, it was observed that many individuals who self-reported allergies after vaccination experienced side effects such as vomiting, headache, gastrointestinal disturbances, and cardiac palpitations compared to non-allergic individuals [19]. Interestingly, in our study, non-atopic individuals mostly reported headaches when compared to atopic individuals in the BNT162 group. In another study evaluating the effect of COVID-19 vaccination on severe asthma, less than 20% of asthmatic patients reported experiencing the common adverse effects such as local pain, weakness, headache, myalgia, arthralgia, and fever after receiving an mRNA vaccine [38]. A different study assessed the side effects after vaccination with BNT162 and CoronaVac in individuals with allergic diseases and observed a predominance of local pain among the adverse effects [39]. The authors reported that local adverse effects such as swelling and redness were noted in allergic patients after receiving the BNT162 vaccine, and these effects tended to be more persistent than in non-allergic individuals [19]. Regarding the CoronaVac vaccine, in one study, no adverse effects were observed in atopic individuals; this was also observed in allergic individuals with respiratory allergies, demonstrating that the vaccine with inactivated virus has less impact on post-vaccine symptoms [40]. Although our study was focused on adverse effects, future studies should be conducted to assess IgG antibodies against SARS-CoV-2 in both atopic and non-atopic groups to improve the knowledge on vaccine efficacy and elucidate any potential differences in immunogenicity.

The non-atopic group comprises patients with negative SPT and/or specific-IgE ELISA results. However, it is noteworthy that some patients with asthma and rhinitis were also included in non-atopic group. This inclusion may initially appear contradictory as they are often associated with allergic sensitization. However, in our cohort, these patients exhibited negative SPT and serology tests outcomes, suggesting the possibility of non-IgE-mediated mechanisms underlying their respiratory symptoms. Non-IgE-mediated mechanisms, such as irritant triggers, proteases, or non-specific airway hyper-responsiveness, could contribute to the asthma and rhinitis symptoms in these individuals. Therefore, while they presented clinical manifestations of allergy, their negative SPT and specific-IgE results indicate a non-atopic endotype. On the other hand, all the individuals from the atopic group had respiratory allergies, but none of them reported severe allergic reactions or anaphylaxis that could be attributed to the vaccines, in contrast to the findings from other studies [17,18]. This observation is remarkable and suggests that despite their atopic status, these individuals did not exhibit a higher propensity for vaccine-related adverse events. These results emphasize the importance of assessing specific IgE levels to understand the underlying mechanisms contributing to vaccine reactions in atopic individuals.

Although allergy is the most prevalent immune disorder, affecting millions of people worldwide, the EACCI (European Academy of Allergy and Clinical Immunology) declared that the COVID-19 vaccine can be administered to allergic patients, except for individuals with allergy to the vaccine components. For BNT162, polyethylene glycol (PEG) is the most hyper-reactive component and ethylenediaminetetraacetic acid (EDTA) is the most allergenic compound found in the ChAdOx1 vaccine [41,42,43]. Our study was focused on investigating the role of respiratory atopy in the adverse effects related to the vaccines. Further studies should be conducted to determine the details of the vaccine pharmacodynamics and other relevant characteristics that could potentially influence the occurrence of adverse effects. This would provide a more comprehensive understanding of the observed adverse effects and their potential associations with specific vaccine formulations.

The total absence of reported allergic or anaphylactic reactions among the 305 individuals, despite a substantial portion of the cohort being comprised of individuals with asthma and rhinitis, highlights the safety of COVID-19 vaccination in this population.

## 5. Conclusions

Overall, our study contributes valuable evidence supporting the safety of COVID-19 vaccination in individuals with respiratory allergies. However, the higher incidence of mild adverse events after receiving the ChAdOx1 vaccine in atopic individuals highlights the necessity of providing guidance on potential adverse effects when administering some categories of COVID-19 vaccines. Healthcare providers should carefully weigh the potential benefits and risks of each vaccine in the context of the medical history and allergy profile of their patients to optimize vaccine safety and effectiveness.

## Figures and Tables

**Table 1:** Demographic and clinical characteristics of the subjects.

Characteristic	Atopic ^1^	Non-Atopic ^1^	Total	*p*
Participants [n (%)]	166 (54.4%)	139 (45.6%)	305	-
Male [n (%)]	63 (37.9%)	37 (26.6%)	100	0.0381
Female [n (%)]	103 (62.1%)	102 (73.3%)	205	
Age (years, mean)	30.7	35.0	-	0.0240
Allergic rhinitis [n (%)]	116 (69.8%)	53 (38.2%)	169	0.4819
Asthma [n (%)]	1 (0.6%)	1 (0.7%)	2	
Allergic rhinitis and asthma [n (%)]	16 (9.6%)	4 (2.8%)	20	
*D. farinae* wheal size (mm, mean)	5.5	0	-	0.0001
*D. farinae*-specific IgE (mean)	2.4	0.7	-	0.0001

^1^ Atopic/non-atopic subjects were classified based on SPT and *D. farinae*-specific IgE levels.

**Table 2:** Adverse effects reported by subjects after first shot of BNT162, CoronaVac, and ChAdOx1 vaccines.

Adverse Effect	BNT162	ChAdOx1	CoronaVac
Local pain	61 (49.6%)	31 (31.9%)	14 (16.5%)
Headache	13 (10.5%)	22 (22.7%)	3 (3.5%)
Muscle pain	14 (11.4%)	30 (30.9%)	5 (5.9%)
Fatigue	20 (16.3%)	13 (13.4%)	5 (5.9%)
Fever	10 (8.1%)	25 (25.8%)	2 (2.3%)
Chill	4 (3.2%)	12 (12.4%)	0 (0%)
Lymph node enlargement	1 (0.8%)	1 (1%)	0 (0%)
Nausea	2 (1.6%)	11 (11.3%)	0 (0%)
Flu-like symptoms	4 (3.2%)	9 (9.3%)	2 (2.3%)

## Data Availability

The raw data supporting the conclusions of this article will be made available by the authors upon request.

## Supplementary Materials

The following supporting information can be downloaded at: https://www.mdpi.com/article/10.3390/vaccines12040408/s1. Figure S1: Comparison among BNT162, ChAdOx1, and CoronaVac COVID-19 vaccines regarding absence or presence of adverse effects reported by subjects; Table S1: Main adverse effects reported by atopic (n = 166) and non-atopic subjects (n = 139) in the first shot of BNT162, ChAdOx1, and CoronaVac vaccines; Table S2: Main adverse effects reported by atopic (n = 78) and non-atopic subjects (n = 45) in the first shot of BNT162 vaccine; Table S3: Main adverse effects reported by atopic (n = 44) and non-atopic subjects (n = 53) in the first shot of ChAdOx1 vaccine; Table S4: Main adverse effects reported by atopic (n = 45) and non-atopic subjects (n = 40) in the first shot of CoronaVac vaccine.
