# Supplementary material for: Distinct Adverse Reactions to mRNA, Inactivated Virus, and Adenovirus Vector COVID-19 Vaccines: Insights from a Cohort Study on Atopic and Non-Atopic Subjects in Brazil
Source: Vaccines (Basel). 2024 Apr 12;12(4):408. doi: 10.3390/vaccines12040408 (PMC11054204; doi:10.3390/vaccines12040408)
Supplement: Supplementary file 1 [file vaccines-12-00408-s001.zip › vaccines-2898422-supplementary.pdf]

Supplementary figures

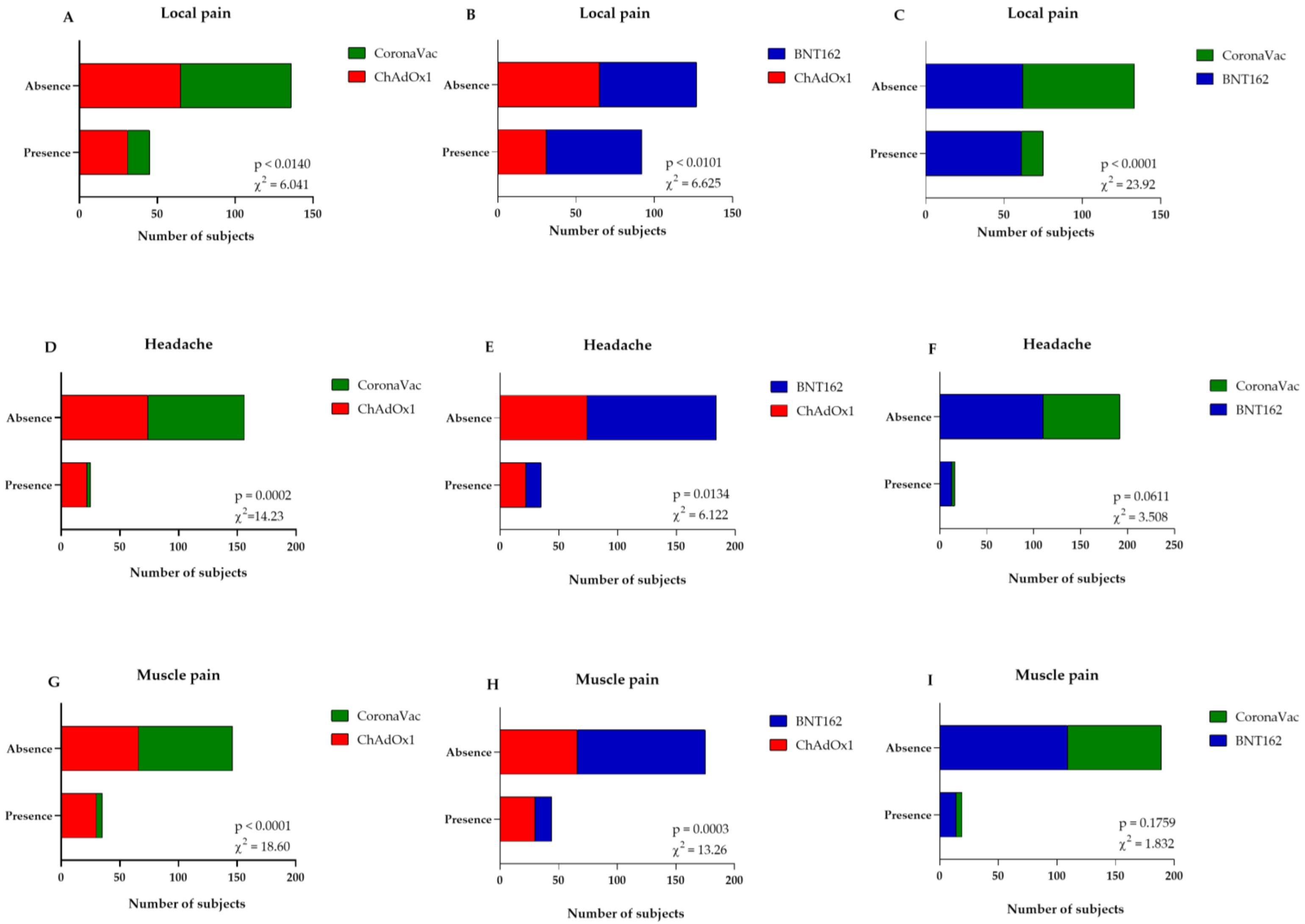

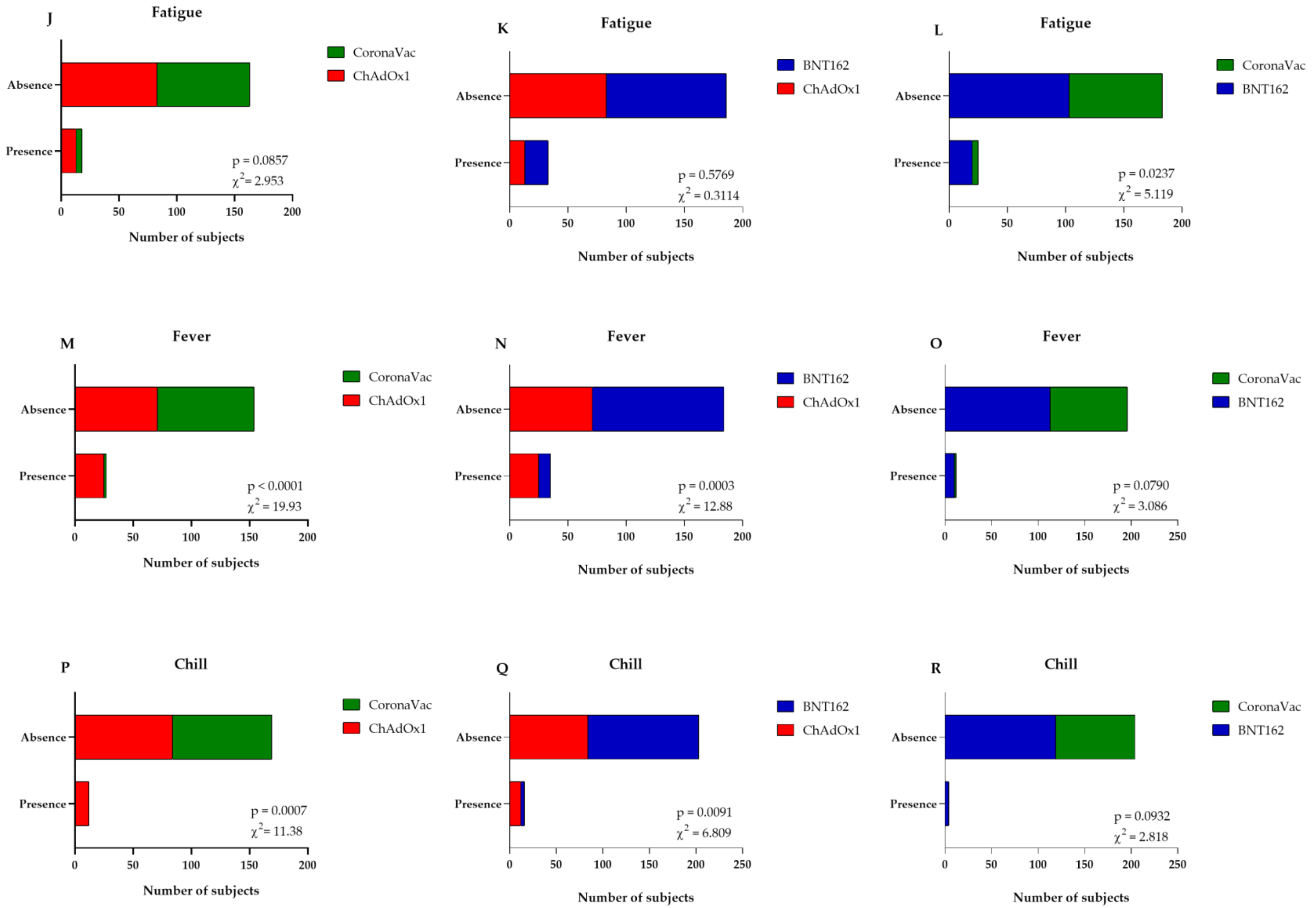

J
Fatigue
CoronaVac
ChAdOx1
Absence
Presence
p = 0.0857
χ2= 2.953
0 50 100 150 200
Number of subjects
K
Fatigue
BNT162
ChAdOx1
Absence
Presence
p = 0.5769
χ2 = 0.3114
0 50 100 150 200
Number of subjects
L
Fatigue
CoronaVac
BNT162
Absence
Presence
p = 0.0237
χ2 = 5.119
0 50 100 150 200
Number of subjects
M
Fever
CoronaVac
ChAdOx1
Absence
Presence
p < 0.0001
χ2 = 19.93
0 50 100 150 200
Number of subjects
N
Fever
BNT162
ChAdOx1
Absence
Presence
p = 0.0003
χ2 = 12.88
0 50 100 150 200
Number of subjects
O
Fever
CoronaVac
BNT162
Absence
Presence
p = 0.0790
χ2 = 3.086
0 50 100 150 200 250
Number of subjects
P
Chill
CoronaVac
ChAdOx1
Absence
Presence
p = 0.0007
χ2= 11.38
0 50 100 150 200
Number of subjects
Q
Chill
BNT162
ChAdOx1
Absence
Presence
p = 0.0091
χ2 = 6.809
0 50 100 150 200 250
Number of subjects
R
Chill
CoronaVac
BNT162
Absence
Presence
p = 0.0932
χ2 = 2.818
0 50 100 150 200 250
Number of subjects

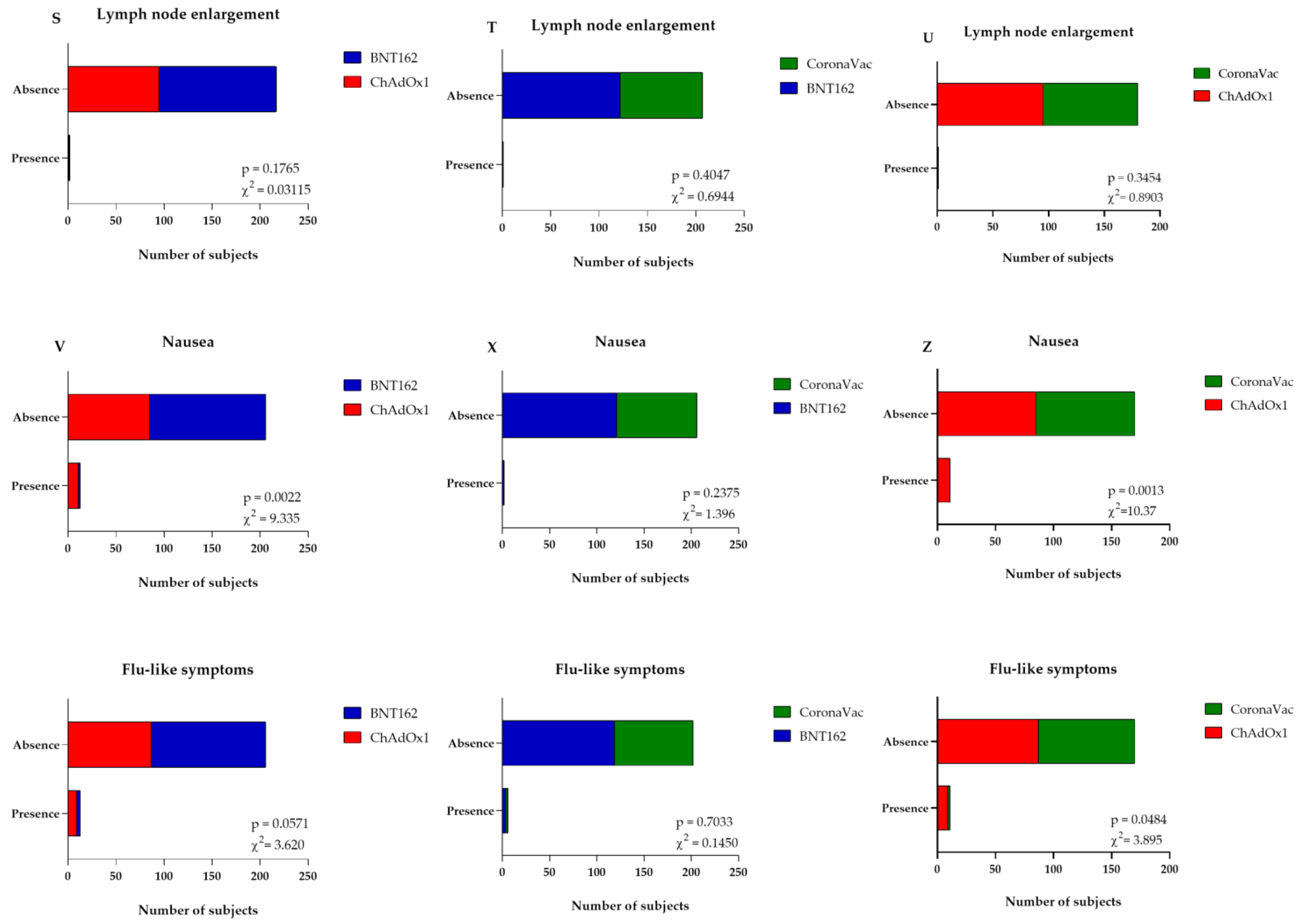

Figure S1. Comparision among BNT162, ChAdOx1, and CoronaVac COVID-19 vaccines regarding absence or presence of adverse effects reported by subjects.

Supplementary tables

Table S1. Main adverse effects reported by atopic (n = 166) and non-atopic subjects (n = 139) in the first shot of BNT162, ChAdOx1, and CoronaVac vaccines.

Adverse effect	Atopic[1]	Non-atopic	p
Local pain	62 (37.3%)	44 (31.9%)	0.3347
Headache	25 (15%)	13 (9.4%)	0.1641
Muscle Pain	31 (18.7%)	18 (13.0%)	0.2109
Fatigue	22 (13.2%)	16 (11.6%)	0.4769
Fever	23 (13.9%)	14 (10.1%)	0.3796
Chill	11 (6.6%)	5 (3.6%)	0.3059
Lymph node enlargement	2 (1.2%)	0	0.5028
Nausea	9 (5.4%)	4 (2.9%)	0.3951
Flu-like symptoms	10 (6.0%)	5 (3.6%)	0.4286

Table S2. Main adverse effects reported by atopic (n = 78) and non-atopic subjects (n = 45) in the first shot of BNT162 vaccine.

Adverse effect	Atopic	Non-atopic	p
Local pain	39 (50%)	22 (48.9%)	0.9999
Headache	10 (12.8%)	3 (6.7%)	0.3703
Muscle Pain	7 (8.9%)	7 (15.6%)	0.3769
Fatigue	10 (12.8%)	10 (22.2%)	0.2079
Fever	3 (3.9%)	7 (15.6%)	0.0362*
Chill	2 (2.6%)	2 (4.4%)	0.6229
Lymph node enlargement	1 (1.2%)	0	0.999
Nausea	1 (1.3%)	1 (2.2%)	0.999
Flu-like symptoms	1 (1.3%)	3 (6.7%)	0.1383

Table S3. Main adverse effects reported by atopic (n = 44) and non-atopic subjects (n = 53) in the first shot of ChAdOx1 vaccine.

Adverse effect	Atopic	Non-atopic	p
Local pain	14 (32.7%)	17 (32.1%)	0.9999
Headache	14 (32.7%)	8 (15.1%)	0.0529
Muscle Pain	20 (46.5%)	10 (18.8%)	0.0044*
Fatigue	8 (18.6%)	5 (9.4%)	0.237
Fever	19 (44.2%)	6 (11.3%)	0.0004*
Chill	9 (20.9%)	3 (5.7%)	0.0316*
Lymph node enlargement	1	0	0.4479
Nausea	8 (18.6%)	3 (5.7%)	0.0589
Flu-like symptoms	7 (16.9%)	2 (3.8%)	0.0734

Table S4. Main adverse effects reported by atopic (n = 45) and non-atopic subjects (n = 40) in the first shot of CoronaVac vaccine.

Adverse effect	Atopic	Non-atopic	p
Local pain	9 (20%)	5 (12.5%)	0.3947
Headache	1 (2.2%)	2 (5%)	0.5991
Muscle Pain	4 (8.9%)	1 (2.5%)	0.3644
Fatigue	4 (8.9%)	1 (2.5%)	0.3644
Fever	1 (2.2%)	1(2.5%)	0.9999
Chill	0	0	0.9999
Lymph node enlargement	0	0	0.999
Nausea	0	0	0.9999
Flu-like symptoms	2 (4.4%)	0	0.4958
